# Multi‐omics and Functional Analyses Identify Cancer‐associated Fibroblast‐derived Insulin‐like Growth Factor 1 as a Stromal Mediator of Lung Adenocarcinoma Progression

**DOI:** 10.1002/mco2.71123

**Published:** 2026-11-05

**Authors:** Jingwen Deng, Jin Sheng, Liangkun You, Qizhen Han, Yueyang Wang, Hong Pan, Yadong Lu, Xiaopeng Cai, Hao Liu, Weidong Han

**Affiliations:** ^1^ Department of Medical Oncology School of Medicine Sir Run Run Shaw Hospital Zhejiang University Hangzhou China; ^2^ Department of Pathology and International Institutes of Medicine The Fourth Affiliated Hospital (Yiwu) Zhejiang University School of Medicine Hangzhou China; ^3^ Department of Pathology Peking Union Medical College Hospital Chinese Academy of Medical Sciences and Peking Union Medical College Beijing China; ^4^ Department of Hepatobiliary and Pancreatic Surgery The Second Affiliated Hospital Zhejiang University School of Medicine Hangzhou China; ^5^ Department of Medical Oncology Zhejiang Cancer Hospital Hangzhou China

**Keywords:** bone metastasis, cancer‐associated fibroblasts, IGF1, lung adenocarcinoma, single‐cell RNA sequencing, tumor microenvironment

## Abstract

Lung adenocarcinoma (LUAD) remains a leading cause of cancer‐related mortality, yet the stromal mechanisms driving rapid disease progression are incompletely understood. Here, we integrated single‐cell RNA sequencing with exploratory whole‐exome sequencing to characterize tumor microenvironment remodeling in a rapidly progressing LUAD patient. Tumor tissue exhibited marked stromal enrichment, with inflammatory cancer‐associated fibroblasts (CAFs) constituting a prominent fibroblast subset. Receptor–ligand and cell‐type‐specific expression analyses identified CAF‐derived insulin‐like growth factor 1 (IGF1) as a potential stromal mediator of LUAD progression. This fibroblast‐specific IGF1 pattern was corroborated by an external single‐cell dataset and by immunofluorescence validation in LUAD tissues with or without bone metastasis. Functionally, recombinant IGF1 enhanced LUAD cell proliferation, migration, and invasion, whereas IGF1 knockdown in CAF‐like cells attenuated these malignant phenotypes in co‐culture models. In vivo, IGF1 knockdown in CAF‐like cells reduced distant metastatic burden, accompanied by alterations in Wnt‐related signaling, extracellular matrix organization, and metastasis‐associated molecules. These findings indicate that CAF‐derived IGF1 contributes to LUAD progression and bone metastatic remodeling through paracrine stromal–tumor communication, highlighting the IGF1/IGF1R axis as a potential stromal‐directed therapeutic target in selected LUAD patients.

## Graphical abstract

Integrated single‐cell RNA sequencing and whole‐exome sequencing characterized a rapidly progressing lung adenocarcinoma (LUAD) patient. Inflammatory cancer‐associated fibroblasts (CAFs) were enriched and identified as a major source of insulin‐like growth factor 1 (IGF1). External single‐cell and clinical validation supported fibroblast‐derived IGF1 in LUAD, especially with bone metastasis. Functionally, CAF‐derived IGF1 promoted LUAD proliferation, invasion, and metastasis. (Created with BioRender.com).

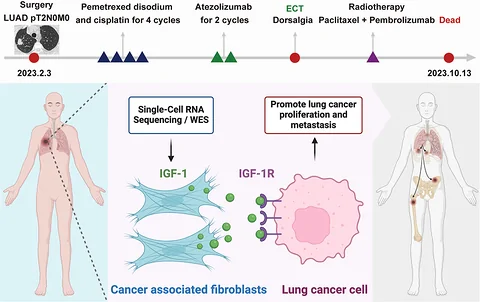

## 1 Introduction

Lung cancer is one of the most prevalent and aggressive malignancies worldwide, with lung adenocarcinoma (LUAD) representing a major pathological subtype. As a subtype of non‐small cell lung cancer, LUAD accounts for around 40% of all lung cancer cases [1]. Many patients are at an advanced stage when first diagnosed, which limits therapeutic options and results in a poor prognosis. Additionally, 10%–30% of patients with early‐stage LUAD experience recurrence after surgical intervention, leading to decreased overall survival [2]. Despite the advent of targeted therapies, treatment failure remains common in advanced‐stage patients, highlighting the urgent need for further investigation of the mechanisms underlying LUAD progression and the development of new therapeutic strategies [3].

One of the most common cell types in the LUAD stroma is cancer‐associated fibroblasts (CAFs), whose regulatory mechanisms in lung cancer remain largely unexplored [4]. Studies have shown that CAFs secrete various cytokines, chemokines, growth factors, and extracellular matrix (ECM) proteins, which are involved in promoting LUAD cell stemness, proliferation, migration, and chemoresistance [5]. CAFs are heterogeneous cell populations, and they can change their features by interacting with surrounding cells, environmental factors such as hypoxia, and the rigidity of their ECM. For example, CAFs not only recruit macrophages and inhibit T cell anti‐tumor immunity but also stimulate the proliferation of vascular endothelial cells [6]. Therefore, while the involvement of CAFs in lung cancer progression is evident, the precise CAF‐derived mediators and their functional roles in LUAD progression warrant further investigation [7, 8].

Insulin‐like growth factor 1 (IGF1), which encodes the IGF1 protein, shares approximately 50% homology with insulin and functions as an endocrine, autocrine, and paracrine growth stimulator to regulate cell growth [9]. Physiologically, IGF1 serves as a major downstream mediator of growth hormone, and its products play a crucial role in mediating the various effects of growth hormone on growth and development. However, increasing epidemiological evidence suggests that high levels of IGF1 are associated with an increased risk of breast, prostate, colon, and lung cancer [9, 10, 11]. Meanwhile, experimental studies have also demonstrated that IGF1 may contribute to lung cancer progression by affecting immunotherapy sensitivity and cancer stemness [12, 13].

IGF1, originally recognized as a hepatokine, is primarily produced in the liver but is also synthesized in most extrahepatic tissues [14]. Studies have found that IGF1 can be secreted by CAFs in a paracrine manner, promoting the progression of colorectal, gastric, and breast cancers [15, 16, 17]. In addition to remodeling the ECM to facilitate tumor cell invasion, CAFs can also regulate malignant phenotypes through growth factor‐mediated paracrine signaling. Yi and colleagues found that CAFs increased the expression and phosphorylation of ANXA2 through the secretion of growth factors HGF and IGF1, which promoted epithelial‐mesenchymal transition and EGFR‐TKI resistance in non‐small cell lung cancers [18]. However, the clinical relevance and cell‐type‐specific expression pattern of CAF‐derived IGF1 in LUAD remain insufficiently defined.

High‐throughput sequencing technologies offer insights into cancer pathogenesis at both the individual and cellular levels, including gene regulation mechanisms, cellular metabolic processes, and molecular response pathways [19]. Single‐cell RNA sequencing (scRNA‐seq) provides a powerful approach for dissecting transcriptomic heterogeneity and identifying cell‐type‐specific signaling programs in the tumor microenvironment (TME). With the development of next‐generation sequencing (NGS), whole‐exome sequencing (WES) can screen the genome‐encoded exome spectrum and identify single‐nucleotide variants, which can help distinguish between hereditary germline mutations and acquired somatic mutations [20]. Therefore, these techniques may guide the diagnosis and treatment of cancers, including LUAD. In the present study, WES was used as an exploratory genomic analysis, whereas the main biological focus was placed on CAF‐derived IGF1 and its functional contribution to LUAD progression.

In this study, we report a LUAD case that progressed rapidly after surgery and medication. Integrated scRNA‐seq and exploratory WES analyses were performed to characterize TME remodeling and genomic alterations in this patient. We found that inflammatory CAFs (CAFinfla) were enriched in tumor tissue and exhibited increased IGF1 expression, suggesting that CAF‐derived IGF1 may participate in LUAD progression through paracrine interactions with tumor cells. This observation was further supported by external single‐cell dataset analysis, clinical tissue validation, and functional experiments. In summary, our study provides valuable insights into the role of CAF‐derived IGF1 in LUAD progression and metastasis, and may help improve the understanding of stromal regulation in rapidly progressing LUAD.

## 2 Results

### 2.1 Clinical Presentation and Disease Course

The patient's medical history and treatment timeline are summarized in Figure 1A. The patient was a 75‐year‐old Asian male with no history of smoking, in whom a right lung mass was detected by chest computed tomography (CT) scan (Figure 1B). After preoperative preparation, thoracoscopic right lung lobectomy with lymph node dissection was performed. The tumor size was approximately 3.6 × 2.4 × 2.2 cm, with visceral pleural involvement, intrapulmonary dissemination, and vascular invasion. The mass was pathologically diagnosed as poorly differentiated adenocarcinoma by hematoxylin and eosin (H&E) staining (Figure 1C). Immunohistochemistry showed positive staining for TTF‐1, CK‐7, and Napsin A, and negative staining for CK5/6, P40, and P63 (Figure 1D, E). The tumor proportion score (TPS) for PD‐L1 expression was 10%, and no alterations in EGFR, ROS1, or ALK were detected. The patient was finally diagnosed with LUAD, staged as pT2N0M0, stage IIA according to the eighth edition of the American Joint Committee on Cancer staging system. The patient's Eastern Cooperative Oncology Group performance status score was 0, with comorbidities of hypertension and gout.

**FIGURE 1:**
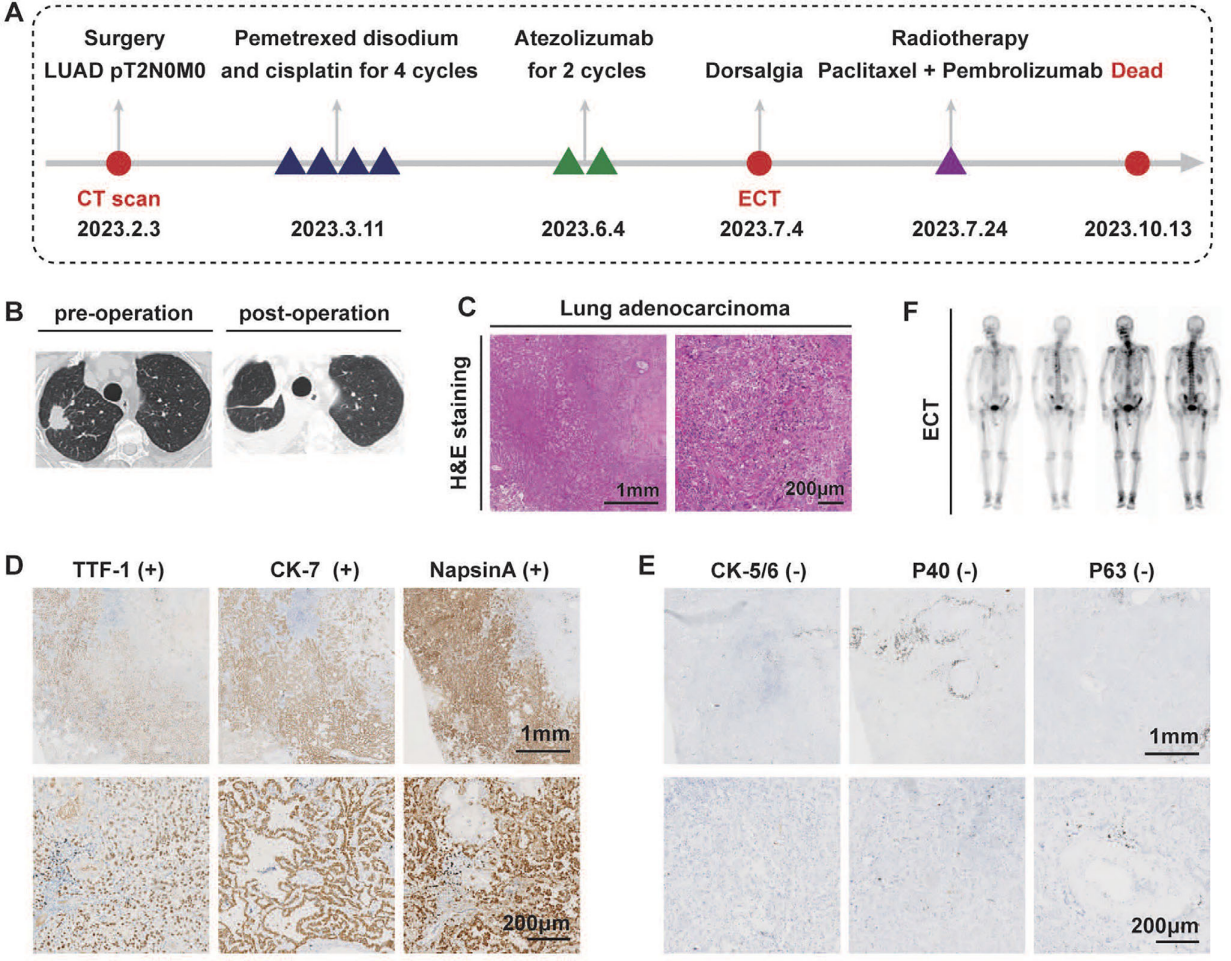
Clinical features of the patient. (A) The medical history and treatment timeline of the patient. (B) Preoperative and postoperative computed tomography (CT) scans of the patient. (C) H&E staining of the tumor tissue. (D) Immunohistochemistry of adenocarcinoma markers TTF‐1, CK‐7, and Napsin A. (E) Immunohistochemistry of squamous cell carcinoma markers CK5/6, P40, and P63. (F) Emission CT (ECT) images of the patient after disease recurrence.

After surgery, the patient received four cycles of pemetrexed disodium plus cisplatin. Subsequent reexamination revealed multiple high‐density shadows in the thoracic vertebrae and left rib, suggesting possible disease recurrence or metastasis. Therefore, the patient received two cycles of atezolizumab as adjuvant therapy. However, the patient experienced increasing back pain following these treatments. A subsequent bone emission CT (ECT) scan revealed multiple areas of increased uptake in the thoracic spine, left posterior ribs, upper segment of the right femur, and left ilium, indicating bone metastasis (Figure 1F). Therefore, the patient received radiotherapy for the thoracic spine and underwent palliative treatment with paclitaxel combined with pembrolizumab. Considering the unexpectedly rapid postoperative progression, intraoperative tumor and adjacent para‐carcinoma tissues were subjected to scRNA‐seq and exploratory WES to characterize TME remodeling and genomic alterations.

### 2.2 Single‐cell RNA Sequencing

With advances in scRNA‐seq, its application to human diseases can enhance our understanding of pathological processes. Therefore, we dissociated patient tumor and para‐carcinoma tissue into a single‐cell suspension and performed scRNA‐seq. After cell filtration, a total of 14,828 high‐quality cells were obtained, consisting of 7079 para‐carcinoma cells (47.7%) and 7749 tumor cells (52.3%). Through unsupervised cluster analysis, we identified 14 distinct clusters (Figure 2A). We annotated these clusters based on existing literature, identifying four primary cell types: endothelial, stromal, immune, and epithelial cells (Figure 2B, C) [21]. Comparing the relative abundance of para‐carcinoma and tumor cells, we observed significant differences in endothelial and stromal cells between the two groups. Tumor tissue contained more stromal cells and fewer endothelial cells (Figure 2D, E). Analysis of intercellular interactions revealed distinct communication patterns between para‐carcinoma and tumor tissues (Figure 2F). In tumor tissue, the interactions between endothelial and stromal cells were stronger, which may be associated with angiogenesis‐related remodeling and ECM reorganization within the TME. Additionally, epithelial cells showed stronger interactions with other cell types in tumor tissue, suggesting enhanced epithelial‐stromal and epithelial‐immune communication rather than directly proving epithelial‐mesenchymal transition or immune evasion. These altered interaction patterns may reflect active TME remodeling during LUAD progression.

**FIGURE 2:**
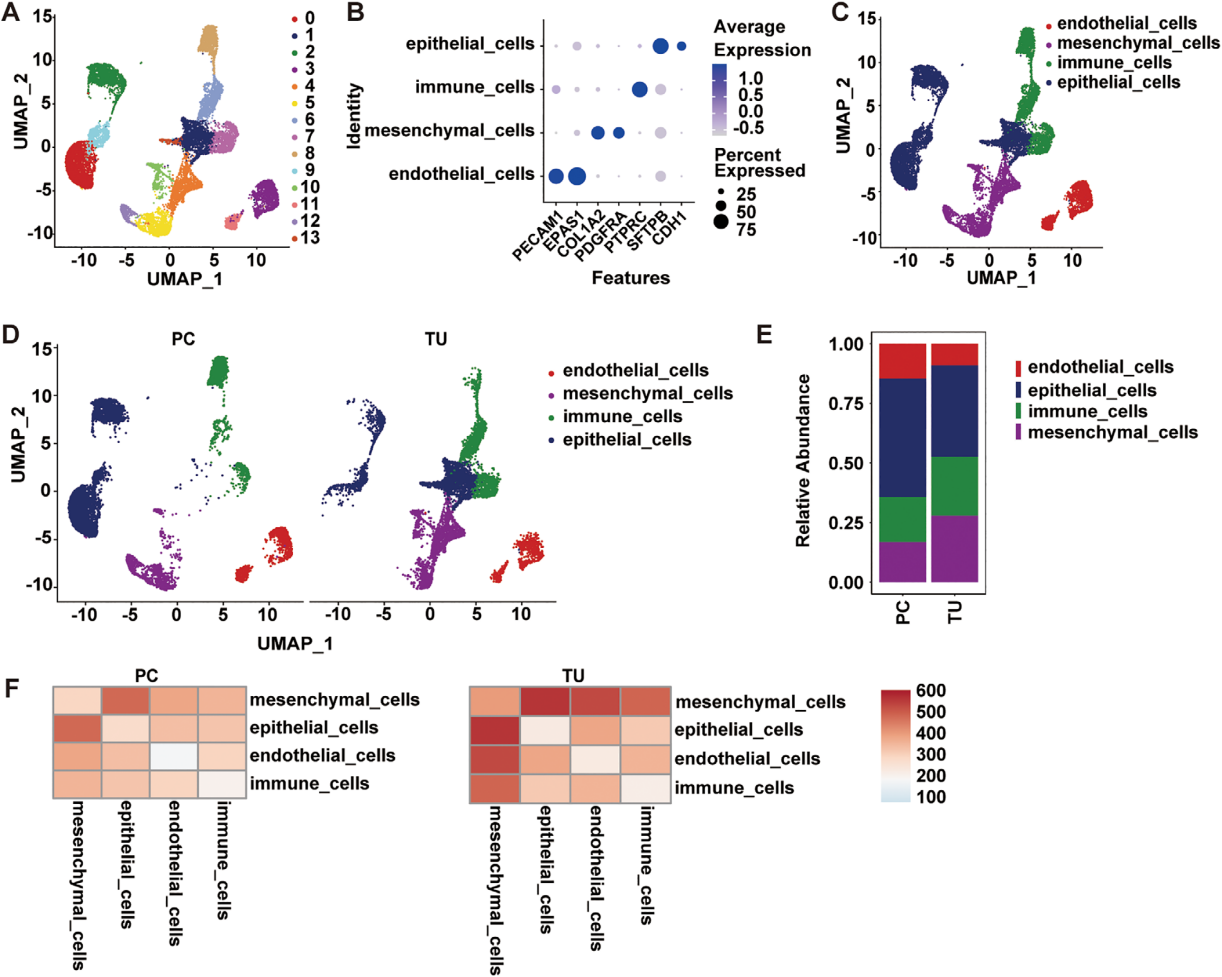
Single‐cell RNA sequencing analysis of para‐carcinoma and tumor tissues. (A) Unsupervised clustering of single cells from para‐carcinoma and tumor tissues. (B) Dot plot showing representative marker genes for the four major cell types. (C) Uniform manifold approximation and projection (UMAP) visualization of the four annotated cell types. (D, E) Relative abundance of the four major cell types in para‐carcinoma and tumor tissues. (F) Cell–cell interaction analysis among the four major cell types in para‐carcinoma and tumor tissues. PC, para‐carcinoma tissue; TU, tumor tissue.

It is well established that fibroblasts represent a major component of lung stromal cells. Therefore, we further classified stromal cells into CAFinfla, normal fibroblasts (NFs), myofibroblastic CAFs (CAFmyo), and endothelial‐to‐mesenchymal transition‐associated CAFs (CAFEndMT) according to previously reported CAF classification frameworks and canonical marker genes [7, 8, 22]. Figure 3A highlights representative marker genes for these fibroblast subtypes, including FAP, COL1A1, and COL3A1 for CAFinfla, as well as ACTA2 and PDGFRB for CAFmyo. Uniform Manifold Approximation and Projection (UMAP) analysis showed that the dominant subgroups, CAFinfla and CAFmyo, exhibited greater heterogeneity, whereas the NF and CAFEndMT subgroups were more concentrated (Figure 3B). Figure 3C illustrates that CAFinfla and CAFmyo were more widely distributed in tumor tissue, suggesting expansion and activation of these fibroblast subpopulations in the TME. Conversely, NFs and CAFEndMT were primarily concentrated in para‐carcinoma tissue. Notably, CAFinfla and CAFmyo were significantly increased in tumor tissue, while NFs were significantly decreased (Figure 3D). Gene Ontology/Kyoto Encyclopedia of Genes and Genomes (GO/KEGG) enrichment analysis of differentially expressed genes (DEGs) in fibroblasts further highlighted functional differences between para‐carcinoma and tumor tissues. For example, CAFmyo was mainly enriched in the PI3K‐Akt signaling pathway, ECM‐receptor interaction, and focal adhesion, whereas CAFinfla was primarily enriched in inflammatory and immune‐related pathways, including the TNF signaling pathway and Th17 cell differentiation (Figure 3E). Moreover, we analyzed receptor‐ligand interactions between fibroblasts and epithelial cells and identified 67 receptor‐ligand pairs (Table S1). Figure 3F shows that the intersection of fibroblast DEGs and receptor‐ligand analysis highlighted IGF1 (Table S1), indicating that CAF‐derived IGF1 may participate in fibroblast‐epithelial communication during LUAD progression. The biological significance of IGF1 in LUAD was further explored in subsequent analyses.

**FIGURE 3:**
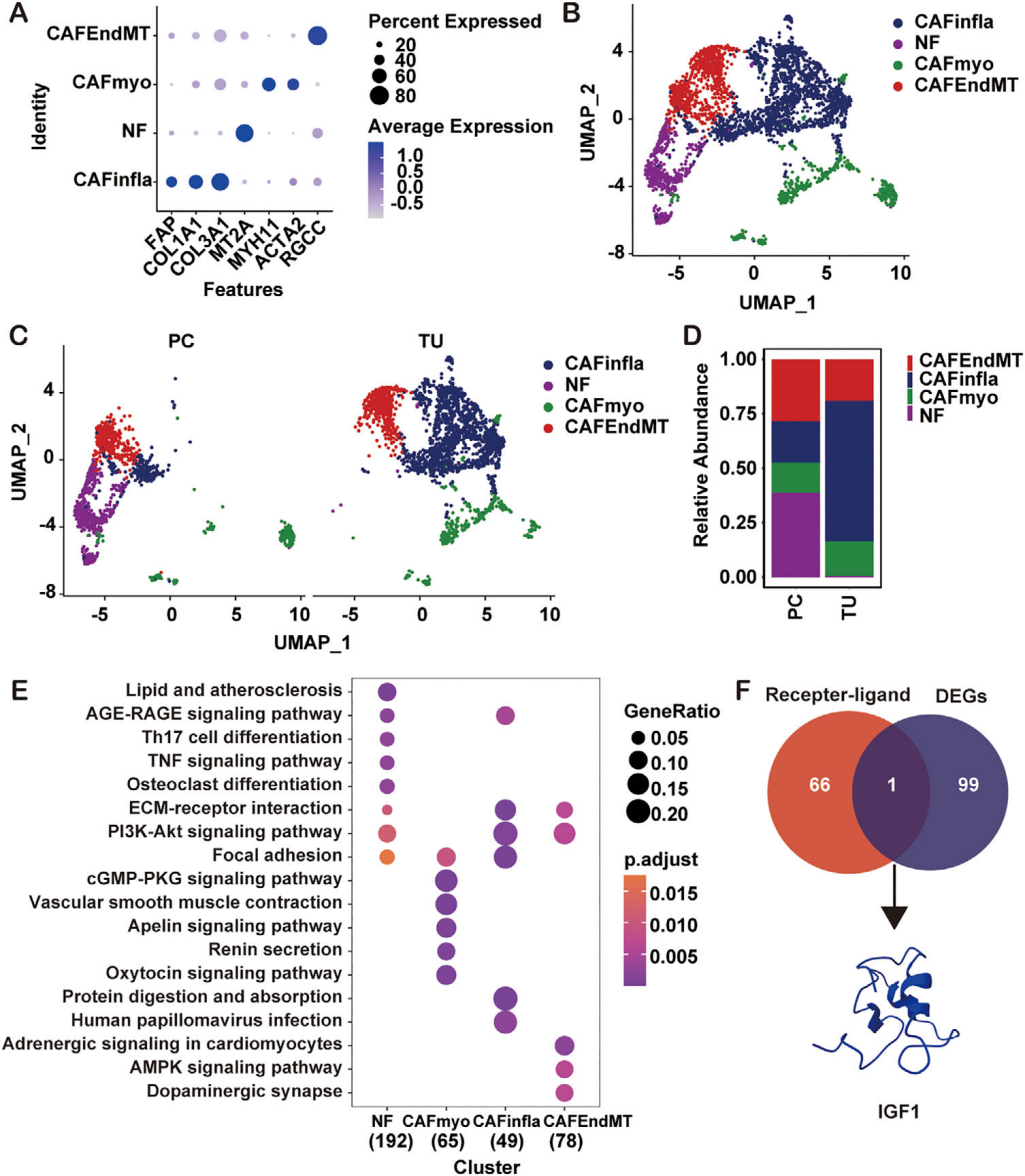
Fibroblast subtype annotation and receptor‐ligand analysis. (A) Dot plot showing representative marker genes for the four fibroblast subtypes. (B, C) Uniform manifold approximation and projection (UMAP) visualization of the four annotated fibroblast subtypes in para‐carcinoma and tumor tissues. (D) Relative abundance of the four fibroblast subtypes in para‐carcinoma and tumor tissues. (E) Gene Ontology/Kyoto Encyclopedia of Genes and Genomes (GO/KEGG) enrichment analysis based on differentially expressed genes in fibroblasts. (F) Venn diagram showing the intersection between fibroblast differentially expressed genes and receptor‐ligand analysis. PC, para‐carcinoma tissue; TU, tumor tissue.

We first analyzed the expression pattern of IGF1 in fibroblast subpopulations from the index patient. As shown in Figure 4A, B, the IGF1 level in CAFinfla was significantly increased in tumor tissue compared with para‐carcinoma tissue. We further quantified IGF1 expression across all major cell types and found that IGF1 was predominantly enriched in stromal cells, especially in tumor‐derived stromal cells (Figure 4C, D). Additionally, the abundance of IGF1 receptor (IGF1R) was relatively higher in multiple cell types within the tumor tissue (Figure 4E, F). These findings suggested that CAF‐derived IGF1 might participate in cell‐cell communication within the TME and potentially contribute to the rapid progression of this patient through paracrine interactions with IGF1R‐expressing cells.

**FIGURE 4:**
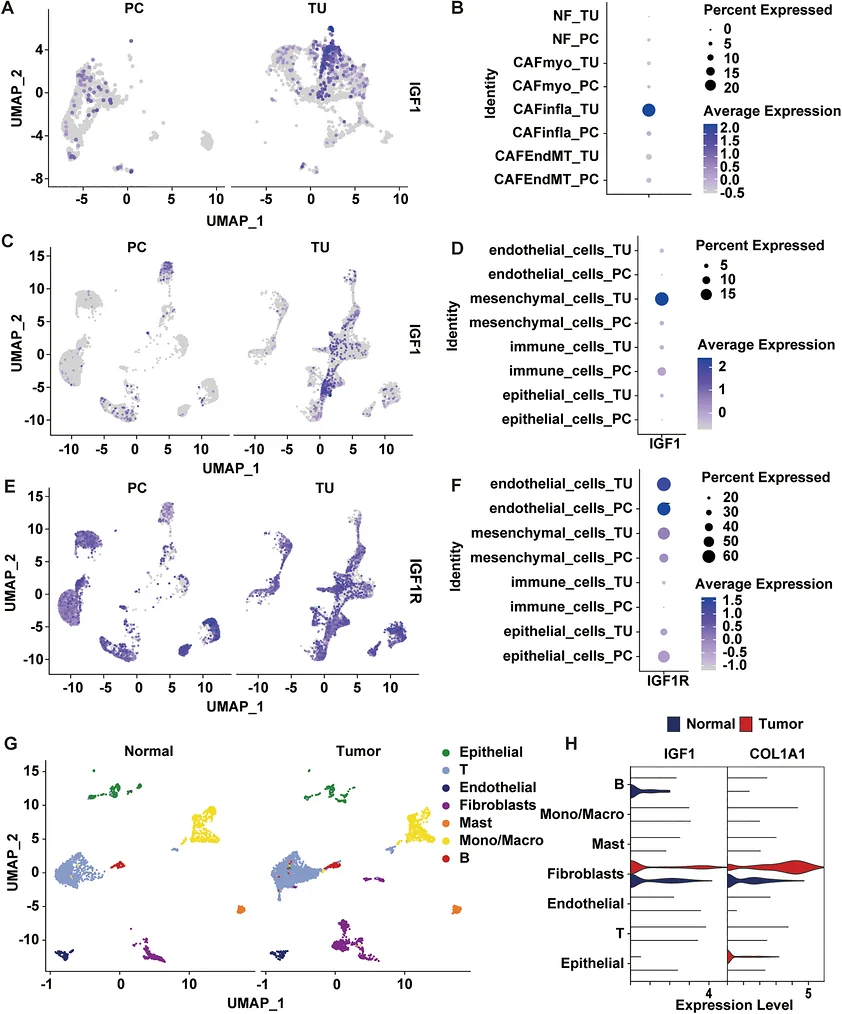
Cell‐type‐specific expression of IGF1/IGF1R and external single‐cell validation. (A, B) Expression analysis of insulin‐like growth factor 1 (IGF1) in fibroblast subpopulations from para‐carcinoma and tumor tissues of the index patient. (C, D) Expression analysis of IGF1 across the major cell types from para‐carcinoma and tumor tissues. (E, F) Expression analysis of insulin‐like growth factor 1 receptor (IGF1R) across the major cell types from para‐carcinoma and tumor tissues. (G) Uniform Manifold Approximation and Projection (UMAP) visualization of annotated cell types in normal and tumor tissues from the external dataset. (H) Violin plot showing IGF1 expression across different cell types in normal and tumor tissues from the external dataset. PC, para‐carcinoma tissue; TU, tumor tissue.

To further address the limitation of single‐patient‐derived single‐cell data, we reanalyzed an external scRNA‐seq dataset containing six normal lung tissues and 12 lung cancer tissues (GSE153935). Major cell types, including epithelial cells, T cells, endothelial cells, fibroblasts, mast cells, monocytes/macrophages, and B cells, were annotated based on canonical marker genes (Figure 4G, H). Consistent with the findings from the index patient, IGF1 was mainly enriched in fibroblasts and showed a higher expression level in tumor‐derived fibroblasts than in fibroblasts from normal lung tissues (Figure 4H). These results support a recurrent fibroblast‐specific pattern of IGF1 upregulation in lung cancer tissues and strengthen the reliability of our single‐patient observation.

Considering that common oncogenic driver alterations, including EGFR, ALK, and ROS1, were not detected in the tumor tissue of the index patient, we further performed exploratory WES to characterize the genomic background of this case. As shown in Figure S1, tumor tissue exhibited more single‐nucleotide polymorphisms and insertion/deletion events than para‐carcinoma tissue. Notably, the IGF1 variants identified in this patient were located in non‐coding regions and were therefore unlikely to directly alter IGF1 protein function. Accordingly, these WES findings were interpreted as exploratory genomic information and are presented in the Supplementary Materials rather than being used as the primary evidence for IGF1 function. The Cancer Genome Atlas (TCGA)‐based exploratory analysis of LUAD samples with IGF1 alterations suggested potential associations with receptor‐ligand activity and Wnt‐related pathways (Figure S1), which provided additional background information but did not replace the cell‐type‐specific and functional evidence. Therefore, the subsequent analyses focused on CAF‐derived IGF1 expression and its biological effects on LUAD progression.

### 2.3 The Functional Evaluation of IGF1 in Lung Cancer

First, we evaluated the direct effect of recombinant IGF1 on lung cancer cells in vitro using A549 and PC‐9 cell lines. As shown in Figure 5A, B, treatment with increasing concentrations of IGF1 (100, 200, and 400 ng/mL) resulted in a dose‐dependent increase in LUAD cell proliferation. In addition, IGF1 treatment significantly enhanced the migration and invasion abilities of LUAD cells in transwell assays (Figure 5C). Based on the above single‐cell findings, we further investigated whether CAF‐derived IGF1 could influence the malignant behaviors of LUAD cells. IGF1 expression was knocked down in human embryonic lung fibroblasts (Helf) cells, which were subsequently induced into CAF‐like cells by transforming growth factor‐β1 (TGF‐β1) treatment (Figure 5D, E). LUAD cells were then co‐cultured with control or IGF1‐knockdown CAF‐like cells. Compared with the control group, co‐culture with IGF1‐knockdown CAF‐like cells markedly reduced the proliferation of LUAD cells (Figure 5F, G). Similarly, the numbers of migrating and invading LUAD cells were significantly decreased after co‐culture with IGF1‐knockdown CAF‐like cells (Figure 5H). To further validate the paracrine effect of CAF‐derived IGF1, we additionally performed direct contact co‐culture assays. Consistent with the above findings, IGF1 knockdown in CAF‐like cells also suppressed the proliferative capacity of LUAD cells under direct contact co‐culture conditions (Figure S2). Together, these results indicate that CAF‐derived IGF1 contributes to the malignant phenotypes of LUAD cells in vitro.

**FIGURE 5:**
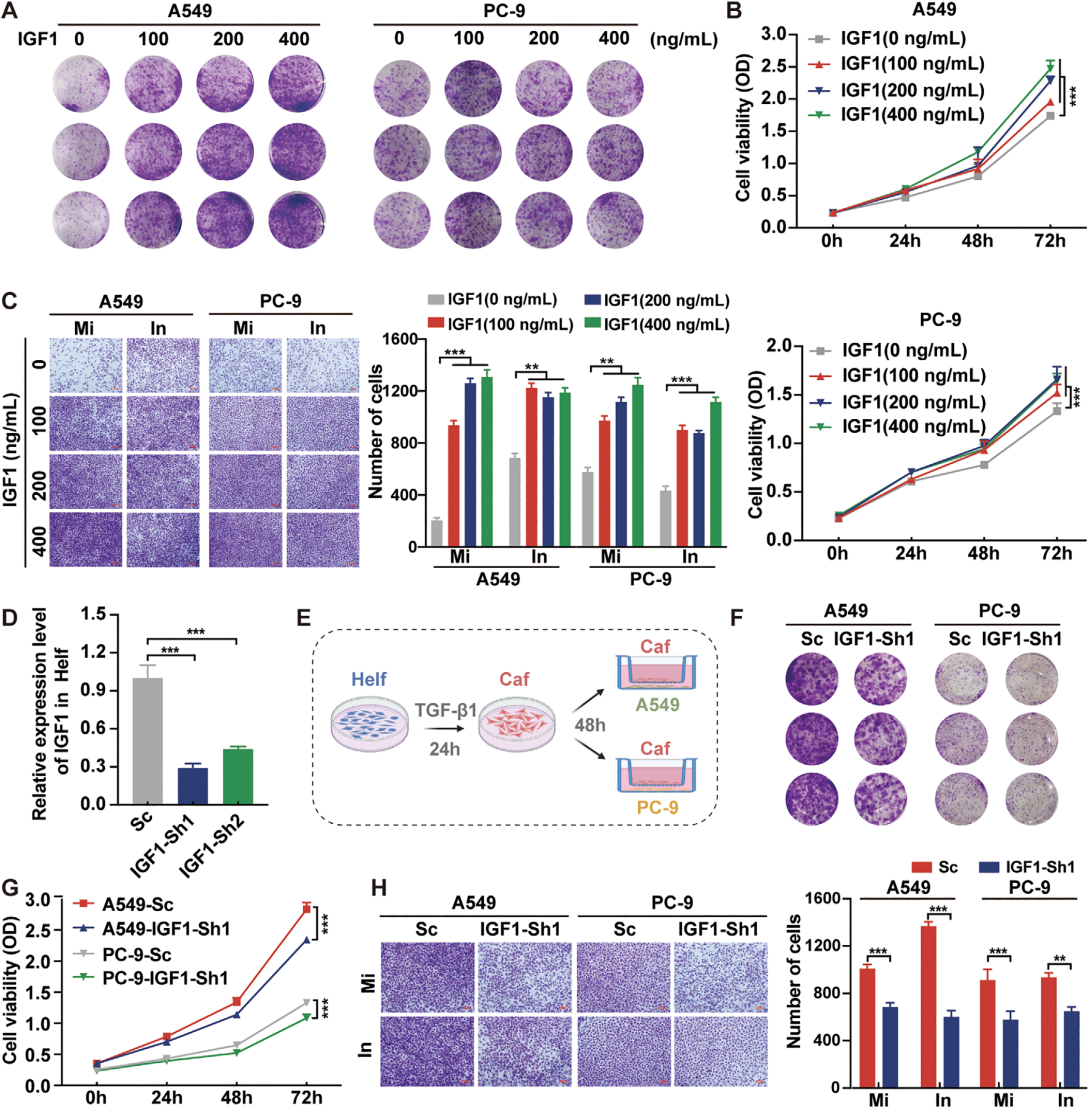
Functional evaluation of insulin‐like growth factor 1 (IGF1) in lung adenocarcinoma (LUAD) cells in vitro. (A, B) Colony formation and cell viability assays of A549 and PC‐9 cells treated with different concentrations of recombinant IGF1. (C) Transwell migration and invasion assays of A549 and PC‐9 cells treated with recombinant IGF1. (D) Knockdown efficiency of IGF1 in Helf cells. (E) Schematic diagram of the co‐culture system. (F, G) Colony formation and cell viability assays of LUAD cells after co‐culture with control or IGF1‐knockdown CAF‐like cells. (H) Transwell migration and invasion assays after co‐culture with control or IGF1‐knockdown CAF‐like cells. Mi, migration; In, invasion. **p < 0.01, ***p < 0.001.

### 2.4 Knockdown of IGF1 in CAFs Suppresses Distant Metastasis of LUAD In Vivo

To validate the role of CAF‐derived IGF1 in LUAD metastasis in vivo, Helf cells stably expressing control short hairpin RNA (shRNA) or IGF1 shRNA were induced into CAF‐like cells and co‐injected with A549 cells into NOD‐SCID mice. Six weeks after injection, mice in the Helf‐Sc group developed obvious metastatic lesions, whereas mice in the Helf‐IGF1‐Sh1 group showed markedly reduced metastatic tumor burden (Figure 6A). Quantitative analysis further confirmed that tumor mass was significantly lower in the Helf‐IGF1‐Sh1 group than in the Helf‐Sc group (Figure 6B). H&E staining showed apparent metastatic involvement in lung tissues and bone lesions in the control group, while these metastatic lesions were substantially reduced after IGF1 knockdown in CAF‐like cells (Figure 6C).

**FIGURE 6:**
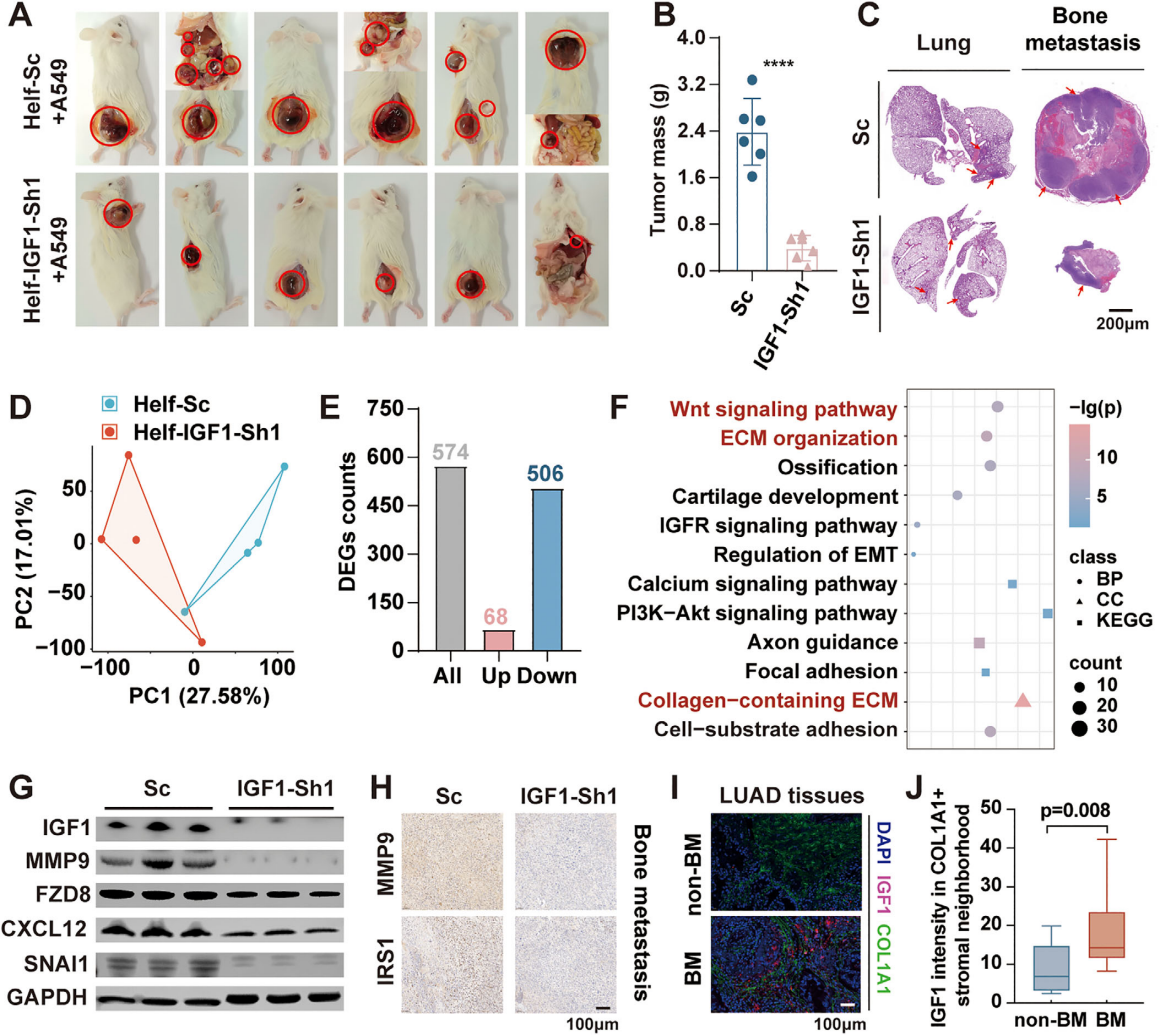
CAF‐derived insulin‐like growth factor 1 (IGF1) promotes lung adenocarcinoma (LUAD) metastasis in vivo and is associated with bone metastasis in clinical samples. (A, B) Representative images and quantification of metastatic lesions in NOD‐SCID mice co‐injected with A549 cells and CAF‐like cells expressing Helf‐Sc or IGF1 shRNA. (C) H&E staining of lung tissues and bone metastatic lesions. (D, E) RNA‐seq analysis of bone metastatic tissues, including experimental design, principal component analysis, and differentially expressed genes. (F) GO and KEGG enrichment analyses of differentially expressed genes. (G, H) Western blot and immunohistochemical validation of metastasis‐related proteins in metastatic lesions. (I, J) Representative immunofluorescence images and semi‐quantitative analysis of IGF1 expression in COL1A1‐positive stromal neighborhoods in LUAD tissues with or without bone metastasis (*n* = 24). DAPI, blue; IGF1, red; COL1A1, green. BM, bone metastasis; non‐BM, non‐bone metastasis. ** *p* < 0.0001.

To further investigate the molecular programs affected by CAF‐derived IGF1 during metastasis, RNA sequencing was performed using bone metastatic tissues from the two groups. Principal component analysis (PCA) showed clear transcriptomic separation between the Helf‐Sc and Helf‐IGF1‐Sh1 groups (Figure 6D). Differential expression analysis identified 574 DEGs, including 68 upregulated and 506 downregulated genes in the Helf‐IGF1‐Sh1 group compared with the Helf‐Sc group (Figure 6E). GO and KEGG enrichment analyses indicated that IGF1 knockdown in CAF‐like cells was associated with changes in metastasis‐related biological processes and signaling pathways, including the Wnt signaling pathway, ECM organization, ossification, IGFR signaling pathway, regulation of EMT, PI3K‐Akt signaling pathway, focal adhesion, and collagen‐containing ECM (Figure 6F).

Western blot analysis further supported these transcriptomic findings. Compared with the Helf‐Sc group, bone metastatic tissues from the Helf‐IGF1‐Sh1 group showed reduced expression of IGF1 and several metastasis‐related proteins, including MMP9, CXCL12, and SNAI1, together with changes in FZD8 expression (Figure 6G). Consistently, immunohistochemical staining showed decreased MMP9 and IRS1 expression in bone metastatic lesions from the Helf‐IGF1‐Sh1 group compared with the Helf‐Sc group (Figure 6H). These results suggest that CAF‐derived IGF1 may promote LUAD metastasis by regulating matrix remodeling, Wnt‐related programs, IGF1‐related downstream signaling, and EMT‐associated molecular changes.

To determine whether this CAF‐associated IGF1 pattern was clinically relevant, we further examined LUAD tissue samples from 24 patients with or without bone metastasis. Representative immunofluorescence staining showed that IGF1 expression was more prominent in LUAD tissues with bone metastasis and was enriched in COL1A1‐positive stromal regions compared with non‐bone‐metastatic LUAD tissues (Figure 6I). Semi‐quantitative analysis confirmed that IGF1 intensity in COL1A1‐positive stromal neighborhoods was significantly higher in the bone metastasis group than in the non‐bone metastasis group (Figure 6J). Together, these in vivo and clinical validation data support that CAF‐derived IGF1 contributes to LUAD metastatic progression, particularly in the context of bone metastasis‐associated stromal remodeling.

## 3 Discussion

Herein, we report a rapidly progressing LUAD case characterized by marked stromal remodeling and early distant metastasis after surgery and systemic treatment. To investigate the potential mechanisms underlying this aggressive clinical course, we integrated scRNA‐seq, exploratory WES, in vitro functional assays, in vivo metastasis experiments, and clinical tissue validation. Based on scRNA‐seq data, we found that stromal cells, particularly CAFinfla, were enriched in tumor tissue compared with para‐carcinoma tissue. Further receptor‐ligand analysis and cell‐type‐specific expression profiling identified IGF1 as a fibroblast‐enriched molecule that may mediate communication between CAFs and tumor cells. This observation was further supported by an external single‐cell dataset and by clinical sample validation, indicating that increased IGF1 expression in fibroblast‐rich stromal regions is not restricted to the index patient.

CAFs are increasingly recognized as functionally heterogeneous components of the TME, with important roles in ECM remodeling, immune regulation, angiogenesis, therapeutic resistance, and metastatic progression [5, 6]. In the present study, CAFinfla and CAFmyo were expanded in tumor tissue, whereas NFs were reduced. Functional enrichment analysis further suggested that these fibroblast subsets were associated with inflammatory responses, ECM‐receptor interaction, focal adhesion, and PI3K‐Akt signaling. These findings are consistent with the concept that distinct CAF subpopulations may exert context‐dependent functions in cancer progression [23]. Importantly, our data suggest that IGF1 is mainly derived from stromal fibroblast populations rather than tumor epithelial cells, supporting a CAF‐centered paracrine mechanism in this rapidly progressing LUAD case.

The IGF1R, the major receptor of IGF1, is widely expressed in normal tissues and solid tumors [24]. Activation of the IGF1/IGF1R axis has been implicated in tumor cell proliferation, survival, migration, stemness, and therapeutic resistance [25, 26]. In our study, IGF1 was enriched in CAFs, while IGF1R was detectable across multiple cell types in the tumor tissue, suggesting the possibility of broad IGF1‐mediated stromal‐tumor communication. Consistently, recombinant IGF1 enhanced the proliferation, migration, and invasion of LUAD cells in vitro, whereas knockdown of IGF1 in CAF‐like cells attenuated these malignant phenotypes in co‐culture models. The additional direct contact co‐culture assays produced consistent results, further supporting the functional contribution of CAF‐derived IGF1 to LUAD cell aggressiveness.

A major extension of the revised study is the in vivo validation of CAF‐derived IGF1 in LUAD metastasis. In the NOD‐SCID mouse model, co‐injection of LUAD cells with IGF1‐knockdown CAF‐like cells markedly reduced visible metastatic lesions and metastatic tumor burden compared with the control group. Transcriptomic profiling of bone metastatic tissues showed that IGF1 knockdown was associated with downregulation of multiple metastasis‐related pathways, including Wnt signaling, ECM organization, PI3K‐Akt signaling, focal adhesion, and ossification‐related processes. Western blot and immunohistochemical validation further supported the involvement of metastasis‐associated proteins and IGF1‐related downstream signaling molecules, including MMP9, FZD8, CXCL12, SNAI1, and IRS1. These results indicate that CAF‐derived IGF1 may promote LUAD metastasis through coordinated regulation of matrix remodeling, Wnt‐related programs, and IGF1R‐associated signaling rather than through a single linear pathway.

The clinical relevance of this CAF‐derived IGF1 pattern was further supported by our independent LUAD tissue cohort. In clinical samples, IGF1 expression was more prominent in LUAD tissues with bone metastasis and showed spatial association with COL1A1‐positive fibroblast‐rich stromal regions. This finding is biologically plausible because CAFs are known to remodel ECM and generate pro‐metastatic niches that facilitate tumor dissemination. Previous studies have also shown that CAF‐derived IGF1 can promote tumor progression in colorectal, gastric, and breast cancers [15, 17]. In non‐small cell lung cancer, CAF‐secreted HGF and IGF1 have been reported to promote epithelial‐mesenchymal transition and EGFR‐TKI resistance through ANXA2‐related signaling [18]. Our study extends these observations by linking CAF‐derived IGF1 to LUAD metastatic behavior, especially bone metastasis‐associated clinical features.

From a therapeutic perspective, the IGF1/IGF1R axis remains a biologically meaningful but clinically complex target. Previous studies have evaluated IGF1R‐targeted strategies, including figitumumab and AXL1717, in non‐small cell lung cancer and other malignancies [27, 28]. Although clinical translation of IGF1R inhibition has been challenging, our findings suggest that patient stratification based on stromal IGF1 activity or CAF‐enriched IGF1 expression may help identify subgroups that are more likely to benefit from therapies targeting this axis. Therefore, rather than considering IGF1 solely as a tumor‐intrinsic oncogenic factor, future studies should also evaluate IGF1 as a stromal mediator within the LUAD TME.

The present study has several strengths. First, it integrates single‐cell transcriptomics, exploratory genomic analysis, in vitro functional assays, in vivo metastasis experiments, and clinical tissue validation, providing a multidimensional assessment of CAF‐derived IGF1 in LUAD progression. Second, the use of an external single‐cell dataset and an independent clinical sample cohort partially addresses the limitation of the initial single‐patient observation. Third, the in vivo metastasis model and downstream molecular validation provide functional evidence supporting the role of CAF‐derived IGF1 in LUAD metastasis. Nevertheless, several limitations should be acknowledged. The initial discovery was derived from one rapidly progressing LUAD patient, and although external datasets and clinical samples were used for validation, larger multicenter cohorts are still needed. In addition, the clinical cohort was limited in size, and longer follow‐up data are required to determine whether CAF‐derived IGF1 has independent prognostic value. Mechanistically, our data support the involvement of Wnt‐related, ECM‐related, and IGF1R‐associated signaling programs, but further studies using pathway‐specific inhibition, rescue experiments, and spatial multi‐omics approaches are needed to define the precise downstream regulatory network.

## 4 Conclusion

This study suggests that CAF‐derived IGF1 may act as an important stromal mediator of LUAD progression and metastasis. By shifting the focus from IGF1 genetic variation to fibroblast‐enriched IGF1 expression and function, our findings provide a more biologically supported framework for understanding stromal regulation in rapidly progressing LUAD. These results may offer new insights into CAF‐centered paracrine signaling and support further investigation of the IGF1/IGF1R axis as a potential therapeutic vulnerability in selected LUAD patients.

## 5 Methods

### 5.1 Sample Collection

Fresh‐frozen surgical specimens, including primary LUAD tissue and matched adjacent normal tissue, were obtained from the index patient at Sir Run Run Shaw Hospital. This study was approved by the Institutional Review Board of Sir Run Run Shaw Hospital (Approval No. 2023.0508), and written informed consent was obtained from the patient. Additional formalin‐fixed paraffin‐embedded LUAD tissue samples were collected for clinical validation, including samples from patients with or without bone metastasis.

### 5.2 Sample Preparation and scRNA‐seq

Single‐cell suspensions with cell viability greater than 80% were washed and resuspended at a concentration of 700–1200 cells/µL for processing using the 10x Genomics Chromium platform (Cosmos Wisdom, Hangzhou, China). cDNA libraries were then constructed within the microfluidic chip according to the manufacturer's protocol. The libraries were sequenced on an Illumina HiSeq or NovaSeq platform using a paired‐end 150‐bp sequencing strategy, with a sequencing depth of at least 20,000 reads per cell.

### 5.3 scRNA‐seq Data Processing

FASTQ files generated from the 10× Genomics platform were aligned to the GRCh38 human reference genome and quantified using Cell Ranger with default parameters. The Cell Ranger output was imported into Seurat (version 4.1.0) using the Read10X function. Doublets were identified for each sample using DoubletFinder based on principal components 1–20, with the following parameters: nExp = 0.08 × (nCells^2^/10,000), pN = 0.25, and pK = 0.09. Cells predicted as doublets were removed before downstream analysis. The filtered single‐cell objects were then merged into an integrated Seurat object, and cell barcodes were renamed to ensure unique cell labels across samples.

Quality control was performed by excluding cells with fewer than 200 or more than 4000 detected genes. After filtering, gene expression data were normalized using the LogNormalize method with a scale factor of 10,000. The top 2000 highly variable genes were identified using the FindVariableFeatures function. Unwanted sources of variation, including UMI counts and mitochondrial gene percentage, were regressed out using the ScaleData function. PCA was then performed based on highly variable genes, and the first 15 principal components were selected for subsequent analysis. Batch effects were corrected using the Harmony package. Cell clustering was performed using the FindNeighbors and FindClusters functions based on a shared nearest‐neighbor graph and the Louvain algorithm, and the clusters were visualized using UMAP. For subclustering analysis, the same workflow, including normalization, variable feature selection, dimensionality reduction, batch correction, clustering, and visualization, was applied.

Differentially expressed genes between clusters were identified using the FindAllMarkers function in Seurat with min.pct = 0.1 and logfc.threshold = 0.25. *p*‐Values were calculated using the non‐parametric Wilcoxon rank‐sum test, and adjusted *p*‐values were computed using the Bonferroni correction.

For fibroblast subtype annotation, cells expressing MT2A and predominantly derived from normal tissues were defined as NFs, whereas the remaining fibroblast clusters were classified as CAFs. Based on canonical marker genes, CAFs were further annotated as inflammatory CAFs (CAF_infla), myofibroblastic CAFs (CAF_myo), and endothelial‐to‐mesenchymal transition‐associated CAFs (CAF_EndMT), characterized by the overexpression of COL1A1/COL3A1, MYH11/ACTA2, and RGCC, respectively.

### 5.4 Cell–Cell Interaction Analysis

CellphoneDB was employed to investigate cell‐cell interactions among all TME components. The input files for statistical analysis consisted of a raw count matrix extracted from the Seurat object and an annotation file specifying cell types. The heatmap_plot function from CellphoneDB and the “Pheatmap” package were utilized to visualize the frequency of interactions between two cell subsets.

### 5.5 Functional Enrichment Analysis

GO enrichment analysis was performed using the R package “clusterProfiler” [29]. GO terms with a *p*‐value<0.05 were considered statistically significant and visualized using the “ggplot2” R package.

### 5.6 Analysis of mRNA Expression

Following standard processing procedures, GEPIA2 was used to analyze RNA sequencing data for tumor and normal samples from TCGA (data retrieved on May 29, 2025) and GTEx projects. The IGF1 gene expression profile was characterized with GEPIA2 across LUAD tumor samples and normal tissues. Gene with |log2(fold change) | > 1 and *p*‐value <0.01 were considered differentially expressed.

### 5.7 WES and Variant Detection

Raw WES data were processed using fastp for quality control, including adapter trimming and removal of low‐quality reads, reads with excessive ambiguous bases, and short fragments after trimming [30]. Clean reads were aligned to the reference genome using the BWA module implemented in Sentieon, followed by BAM sorting with SAMtools, duplicate marking, and base quality score recalibration [31]. Germline SNPs and InDels were identified using the Sentieon Haplotyper/GVCFtyper workflow and annotated with VEP [32]. Structural variants (SVs) and copy number variants (CNVs) were detected using Manta and CNVkit, respectively, annotated with AnnotSV, and visualized with Circos when required [33, 34]. For paired tumor‐normal samples, sample concordance was assessed using BAM‐matcher, and somatic SNPs/InDels were called using Mutect2 with a panel of normals for germline filtering [35, 36]. Somatic variants were annotated with VEP, while somatic SVs and CNVs were analyzed using Manta and CNVkit, respectively.

### 5.8 RNA‐seq Data From TCGA project

Raw count data of 524 LUAD samples were obtained from the TCGA database via the R package ‘TCGAbiolinks’ on May 21, 2025, as in our previous study [37]. After excluding the synonymous mutation of IGF1, 3 samples were defined as mutant samples. The samples from the same tissue were selected as unmutated samples. Using |log2 FC|>1 and *p* < 0.05 as screening criteria, DEGs were further obtained using the R package ‘DESeq2’. Next, GO/KEGG analyses were performed, and the results were visualized with the bioinformatics online tool [38].

### 5.9 The Malignant Behaviors of IGF1 in LAUD

Lung cancer cell lines (A549 and PC‐9) and Helf were purchased from the American Type Culture Collection (Manassas, VA, USA). The cells were cultured in DMEM with 10% fetal bovine serum (Gibco, USA) and grown at 37°C in an atmosphere of 5% CO2.

### 5.10 IGF1 Stimulation Experiment

We treated A549 and PC‐9 cells with different concentrations of IGF1 (100, 200, and 400 ng/mL) (R&D Systems, CAT#291‐G1‐200) and evaluated the proliferation, cell viability, migration, and invasion of cancer cells as in our published article [39].

### 5.11 The Co‐culture Experiments

IGF1‐knockdown Helf cells were generated using a lentivirus‐based shRNA system and then induced into CAF‐like cells with 5 ng/mL TGF‐β1 (MCE, CAT#HY‐P70543). For non‐contact co‐culture, control or IGF1‐knockdown CAF‐like cells were seeded in the lower chamber, and A549 or PC‐9 cells were seeded in 0.4‐µm pore Transwell inserts for 48 h. For direct contact co‐culture, CAF‐like cells and A549 or PC‐9 cells were mixed at a 1:1 ratio and cultured in the same wells for 48 h. After co‐culture, LUAD cells were collected for colony formation, cell viability, migration, and invasion assays as described above.

### 5.12 Animal Studies

All animal experiments were approved by the Institutional Animal Care and Use Committee of Sir Run Run Shaw Hospital (SRRSH2026‐0031). Female NOD‐SCID mice aged 4–6 weeks were obtained from Jiangsu GemPharmatech Co., Ltd. and maintained under specific pathogen‐free conditions. Helf cells stably expressing Helf‐Sc or IGF1 shRNA were induced into CAF‐like cells with 5 ng/mL TGF‐β1. These CAF‐like cells were then mixed with A549 LUAD cells at a 1:1 ratio and injected into the left axilla of NOD‐SCID mice, with a total of 1 × 10^6^ cells per mouse. Approximately six weeks after injection, the mice were euthanized, and visible metastatic lesions, including bone and peritoneal lesions, were dissected, counted, and weighed.

### 5.13 Histological Analysis

For histological evaluation, bone and lung tissues were fixed in 4% paraformaldehyde, paraffin‐embedded, and sectioned. The sections were then stained with H&E for histopathological assessment [40, 41].

### 5.14 RNA Extraction, Library Preparation, Sequencing, and Data Analysis

Total RNA was extracted from bone metastasis tissues using TRIzol Reagent (Invitrogen) [42]. After quality assessment, strand‐specific RNA‐seq libraries were prepared using the Illumina Stranded mRNA Prep Ligation kit (Illumina) according to the manufacturer's protocol and sequenced on an Illumina NovaSeq X Plus platform with a paired‐end 150‐bp sequencing strategy at Cosmos Wisdom Biotech Co., Ltd. (Hangzhou, China). Raw sequencing data were quality‐controlled using fastp. Clean reads were aligned to the reference genome using HISAT2, and gene expression abundance was quantified using RSEM. DEGs between the Helf‐Sc and Helf‐IGF1‐sh1 groups were identified using DESeq2 with thresholds of |log2FC| > 1 and adjusted *p*‐value < 0.05. Functional enrichment analyses of the identified DEGs, including GO and KEGG analyses, were performed using clusterProfiler.

### 5.15 Western Blotting and Immunohistochemistry

For Western blotting, bone metastasis tissues were lysed in RIPA buffer (Beyotime) supplemented with PMSF protease inhibitor. Equal amounts of protein were separated by SDS‐PAGE, transferred onto PVDF membranes (Millipore), and blocked with 5% non‐fat milk [34]. The membranes were incubated overnight at 4°C with primary antibodies against IGF1 (28530‐1‐AP, Proteintech), MMP9 (10375‐2‐AP, Proteintech), FZD8 (55093‐1‐AP, Proteintech), CXCL12 (P011670, EpiZyme), SNAI1 (13099‐1‐AP, Proteintech), and GAPDH (60004‐1‐Ig, Proteintech), followed by incubation with HRP‐conjugated secondary antibodies. For reprobing, membranes were incubated with fast stripping buffer (EpiZyme) and then reprobed as described above. Protein bands were visualized using an Odyssey imaging system (LI‐COR).

### 5.16 Immunohistochemistry

For immunohistochemistry, paraffin‐embedded sections of mouse lung cancer bone metastatic tissues were deparaffinized, rehydrated, subjected to antigen retrieval, and blocked with 5% normal goat serum. The sections were incubated overnight at 4°C with primary antibodies against MMP9 (GB11132, Servicebio) and IRS1 (GB11506, Servicebio), followed by incubation with HRP‐conjugated secondary antibodies. The signals were visualized using DAB substrate, and the sections were counterstained with hematoxylin.

### 5.17 Immunofluorescence

Paraffin‐embedded tissue sections from 24 LUAD patients were deparaffinized, rehydrated, subjected to antigen retrieval, and blocked with 5% normal goat serum. The sections were incubated overnight at 4°C with primary antibodies against IGF1 (28530‐1‐AP, Proteintech) and COL1A1 (GB115707, Servicebio), followed by incubation with fluorophore‐conjugated secondary antibodies. Nuclei were counterstained with DAPI. Fluorescence images were acquired using a fluorescence microscope, and the spatial association between IGF1 expression and COL1A1‐positive fibroblast‐rich stromal regions was evaluated in LUAD tissues with or without bone metastasis.

### 5.18 Statistical Analysis

Data are presented as the mean ± standard deviation. Differences between two groups were analyzed using Student's *t*‐test. A two‐sided *p*‐value < 0.05 was considered statistically significant.

## Author Contributions

All authors contributed to the study conception and design. Material preparation, sample collection, and experimental validation were performed by Jingwen Deng, Jin Sheng, Liangkun You, and Yadong Lu. Data analysis and interpretation were performed by Jingwen Deng, Qizhen Han, Yueyang Wang, Hong Pan, and Xiaopeng Cai. Pathological evaluation and immunostaining analysis were supported by Qizhen Han, Yueyang Wang, and Yadong Lu. The first draft of the manuscript was written by Jingwen Deng and was critically revised by Xiaopeng Cai, Hao Liu, and Weidong Han. All authors read and approved the final manuscript.

## Funding

This work was supported by the National Natural Science Foundation of China (Nos. 82302900 for Cai Xiaopeng, 81702809 for Sheng Jin, and 82173030 for Han Weidong), Zhejiang Provincial Natural Science Foundation of China (No. QN25H160107 for Deng Jingwen) and the Central Guidance for Local Science and Technology Development Funds Project (No. 2024ZY01018 for Han Weidong). The funders played no role in study design, data collection, data analysis, data interpretation, or writing of the report.

## Ethics Statement

This study was approved by the Institutional Review Board of Sir Run Run Shaw Hospital, Zhejiang University School of Medicine (Approval No. 2023.0508), and written informed consent was obtained from the index patient and all participants providing clinical validation samples. All animal experiments were performed in accordance with a protocol approved by the Institutional Animal Care and Use Committee of Sir Run Run Shaw Hospital, Zhejiang University School of Medicine (Approval No. SRRSH2026‐0031), and were conducted in accordance with the guidelines for the care and use of laboratory animals.

## Conflicts of Interest

The authors declare no conflicts of interest.

## Supporting information




**Supporting File 1**: mco271123‐sup‐0001‐SuppMat.docx

## Data Availability

The datasets generated and/or analyzed during the current study are not publicly available because they contain information that could compromise participant privacy. Data may be made available from the corresponding author upon reasonable request and with approval from the Institutional Review Board of Sir Run Run Shaw Hospital.
