# Supplementary material for: Multi‐omics and Functional Analyses Identify Cancer‐associated Fibroblast‐derived Insulin‐like Growth Factor 1 as a Stromal Mediator of Lung Adenocarcinoma Progression
Source: MedComm (2020). 2026 Nov 5;7(11):e71123. doi: 10.1002/mco2.71123 (PMC13740674; doi:10.1002/mco2.71123)
Supplement: Supplementary file 1 [file MCO2-7-e71123-s001.docx]

**Multi-omics and functional analyses identify cancer-associated fibroblast-derived IGF1 as a stromal mediator of lung adenocarcinoma progression**

Jingwen Deng ^1#^, Jin Sheng ^1#^, Liangkun You ^1#^, Qizhen Han ^2^, Yueyang Wang ^3^, Hong Pan ^1^, Yadong Lu ^2^, Xiaopeng Cai ^4*^, Hao Liu ^1*^, Weidong Han ^5*^

1 Department of Medical Oncology, Sir Run Run Shaw Hospital, School of Medicine, Zhejiang University, Hangzhou, Zhejiang 310016

2 Department of Pathology and International Institutes of Medicine, The Fourth Affiliated Hospital (Yiwu), Zhejiang University School of Medicine, Hangzhou, Zhejiang 310058

3 Department of Pathology, Peking Union Medical College Hospital, Chinese Academy of Medical Sciences and Peking Union Medical College, Beijing, 100730, China.

4 Department of Hepatobiliary and Pancreatic Surgery, The Second Affiliated Hospital, Zhejiang University School of Medicine, Hangzhou, Zhejiang 310009;

5 Department of Medical Oncology, Zhejiang Cancer Hospital, No. 1, East Banshan Road, Gongshu District, Hangzhou, P.R. China 310022.

\# Contributed equally.

*Corresponding author: Weidong Han, Hao Liu, Xiaopeng Cai

E-mail: hanwd@zjcc.org.cn; liuhao431@163.com; cxpeng@zju.edu.cn

The authors declare no potential conflicts of interest.

# Supplemental figures

**Figure S1. Exploratory whole-exome sequencing and TCGA-based analysis of IGF1 alterations.** A. Single-nucleotide polymorphism analysis of tumor and para-carcinoma tissues from the index patient. B. Distribution of mutation types in tumor tissue compared with para-carcinoma tissue. C. Insertion/deletion analysis of tumor and para-carcinoma tissues. D. Differentially expressed genes between IGF1-altered and control LUAD samples from TCGA. E–F. GO and KEGG enrichment analyses of differentially expressed genes. G–H. ssGSEA scores of downregulated and upregulated genes projected onto single-cell subsets. PC, para-carcinoma tissue; TU, tumor tissue.

**Figure S2. Direct contact co-culture assay validating the effect of CAF-derived IGF1 on LUAD cells.** A–B. Colony formation and CCK8 assay of A549 and PC-9 cells after direct contact co-culture with control or IGF1-knockdown CAF-like cells. ***p < 0.001.

**Table S1: receptor-ligand analysis in this study**

| **Ligand** | **Receptor** |
| --- | --- |
| ADGRL1 | NRG1 |
| ADORA2A | NAMPT |
| AXL | GAS6 |
| BMP7 | PTPRK |
| BSG | PPIA |
| C5AR1 | RPS19 |
| CADM1 | CADM1 |
| CD226 | NECTIN2 |
| CD44 | FGFR2 |
| CMKLR1 | RARRES2 |
| CSF1 | SLC7A1 |
| CSF1R | CSF3 |
| CSF1R | IL34 |
| CSF3 | CSF3R |
| DLL1 | NOTCH1 |
| DLL1 | NOTCH2 |
| DLL1 | NOTCH3 |
| EGFR | AREG |
| EGFR | BTC |
| EGFR | COPA |
| EGFR | EGF |
| EGFR | GRN |
| EGFR | HBEGF |
| EGFR | MIF |
| EGFR | NRG1 |
| EGFR | TGFA |
| EGFR | TGFB1 |
| EPHA1 | EFNA5 |
| EPHA2 | EFNA5 |
| EPHA3 | EFNA5 |
| EPHB2 | EFNA5 |
| EPOR | KITLG |
| ESR1 | BDNF |
| ESR1 | CXCL1 |
| ESR1 | FGFR2 |
| FGF1 | FGFR2 |
| FGF10 | FGFR2 |
| FGF18 | FGFR2 |
| FGF18 | FGFR3 |
| FGF2 | CD44 |
| FGF2 | FGFR1 |
| FGF2 | FGFR2 |
| FGF2 | FGFR3 |
| FGF2 | FGFRL1 |
| FGF7 | FGFR2 |
| FGF7 | FGFR3 |
| FPR3 | HEBP1 |
| HGF | CD44 |
| HLA-DPB1 | NRG1 |
| IGF1 | IGF1R |
| IL6 | HRH1 |
| JAG1 | NOTCH2 |
| JAG1 | NOTCH3 |
| MST1 | MST1R |
| NCAM1 | BDNF |
| NOTCH1 | CNTN1 |
| NOTCH2 | CNTN1 |
| NOTCH2 | JAG2 |
| NOTCH3 | JAG2 |
| NRG1 | ERBB4 |
| NRG2 | ERBB3 |
| NRG2 | ERBB4 |
| NRG3 | ERBB4 |
| NRP1 | SEMA3A |
| NRP1 | VEGFA |
| NRP1 | VEGFB |
| NRP2 | VEGFA |
| NTN1 | DCC |
| NTRK1 | AIMP1 |
| NTRK1 | COPA |
| NTRK1 | NAMPT |
| NTRK2 | NTN4 |
| PDGFRA | PDGFC |
| PDGFRB | PDGFD |
| PTGIR | GHRL |
| RSPO2 | LGR4 |
| RSPO4 | LGR4 |
| S1PR2 | TAFA5 |
| SEMA4A | PLXND1 |
| SIRPA | CD47 |
| SLIT2 | ROBO1 |
| SLIT2 | ROBO2 |
| TGFB1 | VDR |
| THBS1 | CD36 |
| TIMP1 | FGFR2 |
| TNFSF14 | LTBR |
| TYRO3 | GAS6 |
| UNC5B | NTN4 |
| UNC5C | NTN1 |
| UNC5D | NTN4 |
| WNT2 | SFRP4 |
| WNT2 | WIF1 |
| WNT2B | WIF1 |
| WNT5A | PTPRK |
| WNT5A | ROR1 |
| WNT5A | SFRP4 |
| WNT5A | WIF1 |
| WNT5B | WIF1 |
| WNT9A | SFRP4 |
| WNT9A | WIF1 |
